# Graphene oxide as a protein matrix: influence on protein biophysical properties

**DOI:** 10.1186/s12951-015-0134-0

**Published:** 2015-10-19

**Authors:** Griselle Hernández-Cancel, Dámaris Suazo-Dávila, Axel J. Ojeda-Cruzado, Desiree García-Torres, Carlos R. Cabrera, Kai Griebenow

**Affiliations:** Department of Chemistry, University of Puerto Rico, Río Piedras Campus, San Juan, PR 00931 USA

**Keywords:** Bilirubin oxidase, Graphene oxide, Glycosylation, Structural protein dynamic, Thermostability

## Abstract

**Background:**

This study provides fundamental information on the influence of graphene oxide (GO) nanosheets and glycans on protein catalytic activity, dynamics, and thermal stability. We provide evidence of protein stabilization by glycans and how this strategy could be implemented when GO nanosheets is used as protein immobilization matrix. A series of bioconjugates was constructed using two different strategies: adsorbing or covalently attaching native and glycosylated bilirubin oxidase (BOD) to GO.

**Results:**

Bioconjugate formation was followed by FT-IR, zeta-potential, and X-ray photoelectron spectroscopy measurements. Enzyme kinetic parameters (*k*_*m*_ and *k*_*cat*_) revealed that the substrate binding affinity was not affected by glycosylation and immobilization on GO, but the rate of enzyme catalysis was reduced. Structural analysis by circular dichroism showed that glycosylation did not affect the tertiary or the secondary structure of BOD. However, GO produced slight changes in the secondary structure. To shed light into the biophysical consequence of protein glycosylation and protein immobilization on GO nanosheets, we studied structural protein dynamical changes by FT-IR H/D exchange and thermal inactivation.

**Conclusions:**

It was found that glycosylation caused a reduction in structural dynamics that resulted in an increase in thermostability and a decrease in the catalytic activity for both, glycoconjugate and immobilized enzyme. These results establish the usefulness of chemical glycosylation to modulate protein structural dynamics and stability to develop a more stable GO-protein matrix.

**Electronic supplementary material:**

The online version of this article (doi:10.1186/s12951-015-0134-0) contains supplementary material, which is available to authorized users.

## Background

Understanding the effect of nanomaterials on protein functional and biophysical properties is of fundamental importance in the area of bio-nanotechnology. Enzymes are protein molecules involved in all processes essential for life, but their function depends on the integrity of their secondary and tertiary structure. Unfortunately enzymes can undergo conformational changes due to interference or disruption of the non-covalent interactions stabilizing them (electrostatic interactions, hydrogen bonds, and van der Waals forces). Furthermore, the structural dynamics of protein molecules is a significant component that regulates the proper catalytic function, which until now has largely not been considered during the discussion of the effect of nanomaterials on proteins. Previous studies usually correlate the loss in catalytic activity with changes in the protein structure [1–3], however here we provide evidence that protein dynamics is affected when the enzyme is covalently attached to graphene oxide (GO) nanosheets. Substrate recognition requires protein flexibility because it facilitates conformational rearrangements at the substrate-binding region [4, 5]. Hence, any change in protein dynamics caused by the nanomaterial will affect protein catalytic activity. The influence of structural dynamics on enzyme kinetics has been studied in detail in our laboratory [6–8].

GO has been used as a matrix for enzyme immobilization in different biotechnological applications, such as biosensors [9, 10], biofuel cells [11], cellular imaging [12], gene and drug delivery [13, 14], among others [15–17]. Therefore, the influence of GO on protein structure and its function should be addressed to obtain information that is pertinent across multiple disciplines. In addition to the fundamental information, it is imperative to implement protein stabilization strategies during the immobilization procedure in order to extend the final product shelf life. GO sheets provide large surface areas with surface oxygen-containing groups which have been functionalized with polyethylene glycol (PEG) [18], monolayers of the *N*-hydroxysuccinimidyl ester tripod (NHS-tripod) [19], cationized bovine serum albumin (cBSA) [1], and concanavalin A (Con A) [20] to improve the structure, activity, and stability of enzymes. Herein, we explore the concept of protein engineering through the attachment of polysaccharide chains, also known as glycans, to the ε-amino group of lysine residues. This strategy has been used previously by our group and other researchers to enhance the stability of proteins in pharmaceutical formulations, biosensors, and others biotechnological applications [7, 8, 21–26]. Chemical glycosylation of proteins has proven to be an excellent alternative to modulate protein properties; hence, we are seeking to extend those findings to applications based on carbon nanomaterials.

Several studies have investigated the potential effect of GO on proteins, such as, glucose oxidase (GOD) [1, 27], horseradish peroxidase (HRP) [1–3], oxalate oxidase (OxOx) [2], lysozyme [1, 3], cytochrome c (Cyt C) [1], catalase [1], bovine serum albumin (BSA) [1], chymotrypsin [18], trypsin [18], and proteinase K [18]. Zang et al. found that HRP and lysozyme were immobilized on GO sheets through electrostatic interactions if the pH level was below the isoelectric point (pI), but if the pH level was above the pI they suggested that hydrogen bonds interaction prevailed [28]. In a subsequent publication, they performed different reduction degrees of GO and showed that above the enzyme’s pI, hydrophobic interactions were responsible for enzyme loading [2]. They also found that when the phosphate buffer concentration (ionic strength) increased from 10 to 100 mM the amount of immobilized enzyme increased.

**Fig. 1:**
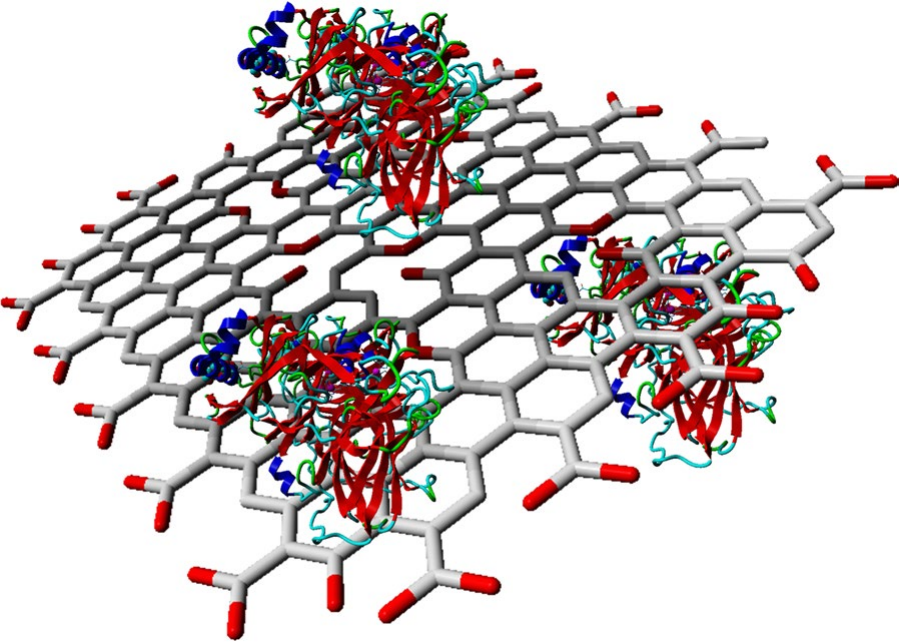
Schematic model of BOD immobilized onto graphene oxide nanosheets (PDB-2XLL)

In the present work we investigated the effect of GO nanosheets on BOD catalytic activity, structure, dynamics, and thermal stability (Fig. 1). BOD is a multi-copper oxidase that has been used in several biotechnological applications, such as, biosensors [29, 30], biofuel cells [31–33], and other environmental and industrial applications [34, 35]. Due to the wide range of biotechnological applications, BOD was selected as our model enzyme. This is the first time that BOD was chemically glycosylated and the effect on protein structural dynamics caused by graphene oxide nanosheets was investigated. We show that compared to native BOD, glycosylation of BOD produced a decrease in the structural dynamics while the thermostability increased. Likewise, when the glycosylated BOD was immobilized on GO nanosheets, the decrease in structural dynamics and the increase in thermostability were even more pronounced. These data demonstrate the potential application of chemically glycosylated proteins to produce highly stable biofunctionalized GO nanosheets for bio-nanotechnology applications.

## Results and discussion

Despite the progress made in materials science combined with biotechnology, there still is a lack of fundamental knowledge regarding the effect of nanomaterials, such as graphene oxide, on biophysical protein properties. Also, the potential applications of enzymes within a manifold of bio- and nanotechnological areas have been hampered by stability issues and remain a critical factor that has to be overcome. Herein, we performed a comprehensive study on how protein biophysical properties (e.g., function, structure, dynamics, and thermostability) were affected as the result of the immobilization process. We also explore the possibility to chemically glycosylate the enzyme to retain protein stability without losing its functionality.

### Characterization of BOD-GO bioconjugates

The enzyme immobilization in GO was carried out as described in the experimental section. The enzyme loading was determined from the supernatant. The estimated concentration of immobilized protein, both, adsorbed or covalently attached to GO, was 0.2 mg mL^−1^. Zhang et al. showed that when reducing the oxidation degree of GO the loading of the HRP increases from 0.1 to 1.31 mg mL^−1^ [2]. This result suggests that our GO is not fully oxidized due to the amount of enzyme that was immobilized on the GO nanosheets. Refer to the supporting information (Additional file 1) for more detail about the synthesis and characterization of GO. The zeta-potential can provide additional evidence of the formation of the BOD-GO bioconjugates (Table 1). GO in 0.1 M PBS pH 7.4 solution shows a zeta-potential of −23 ± 1 mV in agreement with previously reported potentials at ~pH 7 [27]. According to the manufacturer, the isoelectric point of BOD is 4.1, which implies that under the experimental conditions BOD exists as a polyanion. Native BOD in 0.1 M PBS pH 7.4 at a concentration of 0.2 mg mL^−1^ shows a zeta-potential of −6 ± 1 mV. After the immobilization a positive shift of approximately 8 mV was observed, indicating that a considerable change in the surface charge of GO occurred due to the enzyme immobilization.

**Table 1:** Zeta-potential values of graphene oxide after the oxidation process, native and glycosylated BOD, and bioconjugate formation

Sample	Solvent	Z potential
GO	0.1 M PBS pH 7.4	−23 ± 1
BOD		−6 ± 1
Dex-BOD		−4 ± 0.9
BOD-GO-A		−16 ± 1
BOD-GO-C		−15.5 ± 0.8
Dex-BOD-GO-C		−13.6 ± 0.6

**Fig. 2:**
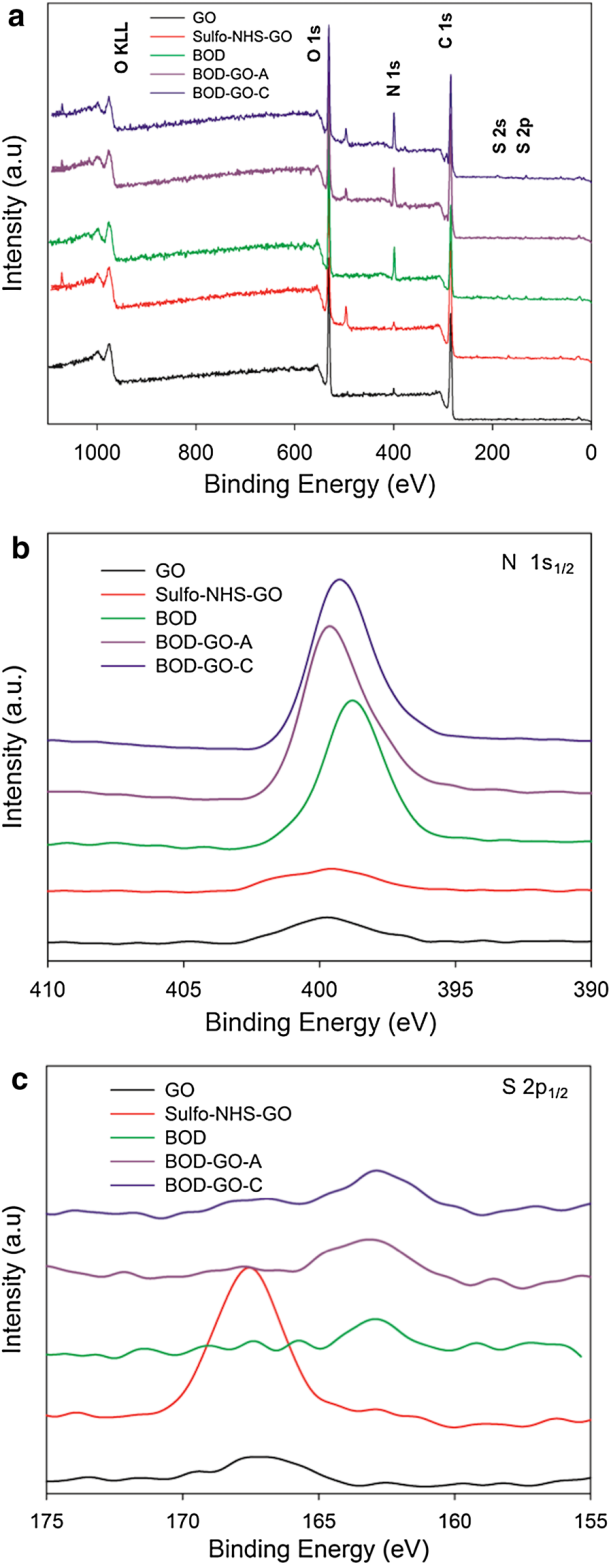
X-ray photoelectron spectroscopy binding energy survey (**a**) and high resolution spectra of N 1s_1/2_ (**b**) and S 2p_1/2_ (**c**) binding energy region for GO, sulfo-NHS-GO, BOD, BOD-GO-A, and BOD-GO-C. All spectra were background corrected and vertically displaced for ease of visualization

X-ray photoelectron spectroscopy (XPS) was used to verify the activation of GO with sulfo-NHS and the immobilization of BOD on GO. The appearance of an S 2p_1/2_ binding energy signal at 168 eV corresponding to the sulfite anion indicates the formation of the reactive intermediate upon activation of the GO with sulfo-NHS (Fig. 2b). As expected, the sulfite anion signal disappears upon enzyme immobilization suggesting the formation of a covalent bond between GO and BOD. The only binding energy peak that remained corresponds to the single cysteine residue in BOD at 163 eV (Fig. 2b) [36]. This peak (163 eV) was also observed in the BOD-GO-A bioconjugate indicating the presence of the protein in GO. The XPS high-resolution spectra of N 1s_1/2_ were used to corroborate the EDC/sulfo-NHS intermediate and the presence of the immobilized protein on GO (Fig. 2b). We observed the presence of two different N atom populations; one at 400 eV attributed to the protonated amine of EDC and the second at 403 eV indicating the presence of the nitrogen of the sulfo-NHS in accordance with previously reported data [22]. An intense N binding energy peak at 398 eV corresponding to the N 1s_1/2_ orbital was observed in the high-resolution spectra of the protein (Fig. 2a). After the immobilization, the nitrogen peak of the protein shifted slightly to higher binding energies (401 eV) indicating a change on the environment of the protein as a result of the interaction with GO. In conclusion, the XPS data confirmed the individual bioconjugate construction steps.

**Fig. 3:**
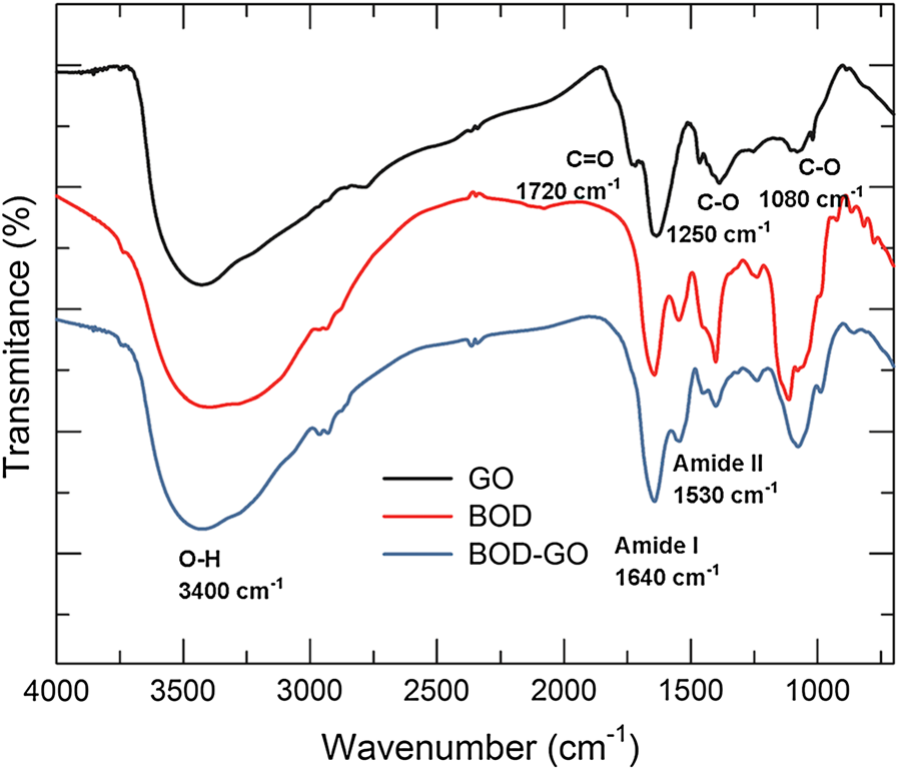
FT-IR spectra of GO, BOD, and BOD-GO

From the FT-IR spectra we also determined the presence of BOD in GO. Figure 3a shows the characteristic FT-IR spectrum of GO. The amide I mode (1640 cm^−1^) largely corresponds to the C=O stretching vibration and the amide II (1530 cm^−1^) mode has N–H bending and C–N stretching vibration contributions (Fig. 3c) demonstrating the successful immobilization of BOD in GO. It is interesting to note that the peak that corresponds to carboxylic acid groups (C=O) at 1720 cm^−1^ disappears as a result of this immobilization, confirming the coupling of BOD in GO.

### Chemical glycosylation of bilirubin oxidase

**Fig. 4:**
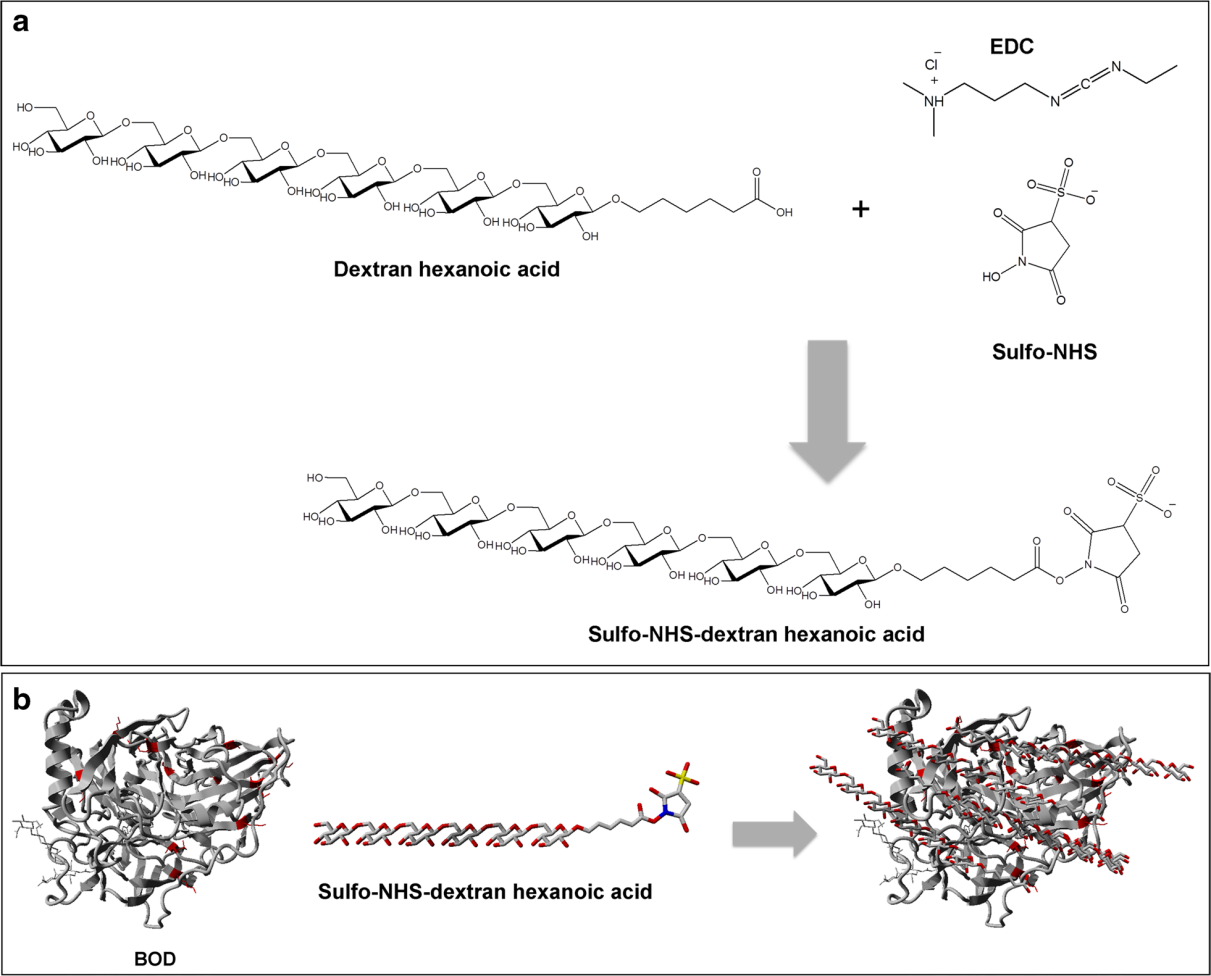
Activation of dextran hexanoic acid with EDC/sulfo-NHS (**a**). Scheme representing the general reaction between BOD and activated dextran to obtain the glycoconjugate (**b**). BOD (PDB-2XLL): solvent accessible lysine residues are shown in *red*. Sulfo-NHS-Dextran: *red* oxygen, *blue* nitrogen, *yellow* sulfur, *grey* carbon

**Fig. 5:**
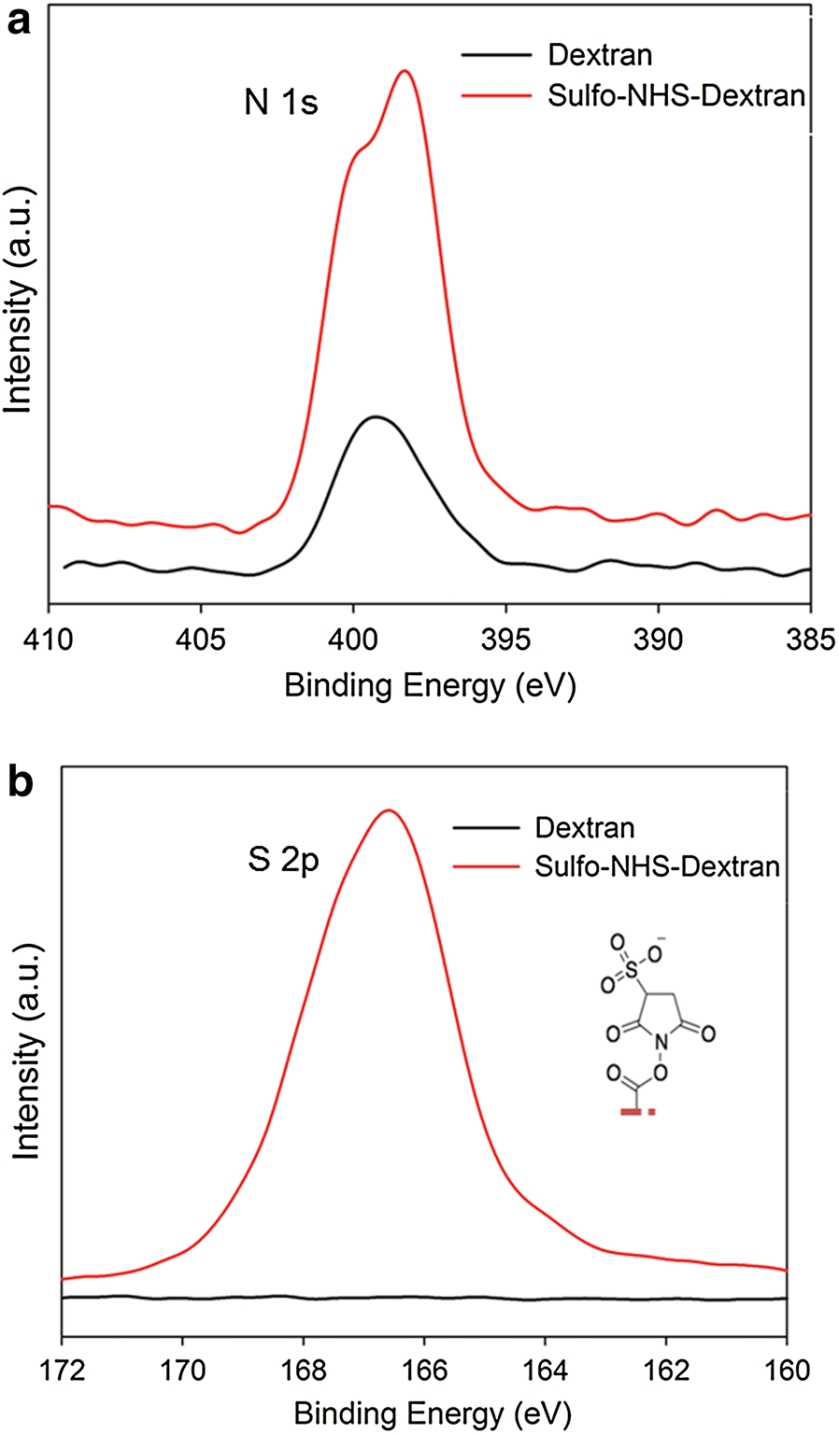
High-resolution X-ray photoelectron spectroscopy of N 1s_1/2_ (**a**) and S 2p_1/2_ (**b**) binding energy region acquired from dextran and dextran activated with sulfo-NHS. All spectra were background corrected and vertically displaced for ease of visualization

Chemical glycosylation is a useful approach to modulate protein structural dynamics without altering the amino acid composition, thus allowing the study of its impact on the fundamental biophysical properties [7]. Another benefit of protein glycosylation is the increase in protein stability, which results in a more robust protein that can better resist denaturing conditions encountered during the production of biotechnological devices and pharmaceutical formulations, extending the useful life of the final product. To form the glycolconjugate we attached dextran (1 kDa) by suitable linker chemistry to the lysine and amino-terminal groups of BOD, which has 11 solvent accessible amino residues. First, dextran hexanoic acid was activated to form sulfo-NHS-Dex (Fig. 4a). Then, the sulfo-succinimidyl group was used us to couple the glycan to the protein (Fig. 4b). XPS was used to confirm dextran activation. Figure 5a, b shows the XPS high-resolution spectra of N 1s_1/2_ and S 2p_1/2_ binding energy regions, respectively. The results demonstrate two different N species that correspond to EDC/Sulfo-NHS with an intense peak at 399 and 401 eV (Fig. 5a). The XPS peak at 166 eV corresponding to the sulfur atom (S 2p_1/2_) indicates the sulfo-NHS-Dex formation.

The TNBSA method was used to determine the amount of covalently attached dextran [37]. Our results demonstrate that 5.7 ± 0.5 lysine residues of BOD were glycosylated and the conjugate is thus referred to as Dex-BOD. To examine if the glycosylation process produces a shielding effect, hence decreasing the formal charge of the protein, we performed a zeta-potential analysis. Table 1 shows the average zeta-potential of the native (6 ± 1 mV) and the glycosylated BOD (4.9 ± 0.9 mV) in 0.1 M PBS pH 7.4 at 25 °C. These results show a decrease in the formal charge of the protein upon glycosylation due to the attachment of the glycan to the protein surface.

### Specific activity and kinetic parameters

Lyophilization is a common procedure used to formulate proteins for long-term storage. It is well know that protein stability during freeze-drying is affected due to stresses that arise from the low temperature, formation of ice, and dehydration [38]. For this reason we decided to study how this step during sample preparation would affect specific activity of native BOD, denoted as Lyo-BOD (Table 2). The Lyo-BOD was dissolved in water, frozen in liquid N_2_, and lyophilized for 48 h, then reconstituted with 0.1 M PBS pH 7.4. We found that the catalytic properties of Lyo-BOD and Dex-BOD decreased significantly while immobilized BOD-GO-A and BOD-GO-C remained highly active. But in comparison with the adsorbed bioconjugate, the BOD-GO-C showed a decrease in the catalytic activity, implying that covalent binding in some way affects the catalytic performance of this bioconjugate. Although the catalytic activity was affected, the *K*_*m*_ values of the samples remained essentially the same, indicating that they all had a similar substrate affinity.

**Table 2:** Comparison of catalytic parameters derived from the oxidation of ABTS by BOD

	Specific activity (U mg^−1^)	*K*_*m*_ (mM)	*k*_*cat*_ (s^−1^)	*k*_cat_/K_m_
BOD	3618 ± 28	29.1 ± 0.6	385 ± 18	13.2 ± 0.5
Dex-BOD	1841 ± 25	26.8 ± 0.6	171 ± 10	6.4 ± 0.3
Lyo-BOD	1908 ± 23	25.2 ± 0.5	195 ± 11	7.8 ± 0.6
BOD-GO-A	3302 ± 29	30.2 ± 0.6	325 ± 23	10.7 ± 0.7
BOD-GO-C	2435 ± 28	27.8 ± 0.9	230 ± 12	8.3 ± 0.5
Dex-BOD-GO-C	1321 ± 27	28.4 ± 0.5	137 ± 8	4.8 ± 0.1

The catalytic activity of covalent bioconjugate (BOD-GO-C), in comparison to BOD-GO-A, was significantly affected. Therefore, we decided to examine if chemical glycosylating could enhance the performance of immobilized BOD the resulted covalent bioconjugate. We observed that Dex-BOD-GO-C also showed a decrease in the specific activity that was not caused by the immobilization, rather by the glycosylation process. We also found a reduction in the turnover rate (*k*_*cat*_) for the Lyo-BOD, Dex-BOD, and therefore Dex-BOD-GO-C. Although the decrease in *k*_*cat*_ value was more significant for the previous samples, there was a minimal change in the adsorbed and covalent bioconjugates. These results suggest that GO does not have a significant influence on the catalytic activity of the glycosylated enzyme. However, the lyophilization and glycosylation process caused a reduction in catalytic efficiency (*K*_*m*_*k*_*cat*_^−*1*^) of the protein in comparison with that of the native BOD. Proteins are dynamical systems and their catalytic activity requires a balance between flexibility and stability. Enzymes must be stable enough to retain their native tertiary structure, but dynamic enough to perform the substrate binding and the subsequent product release [8, 39]. Previous research has shown that protein dynamics are affected by the glycosylation process [7]. To further understand if the catalytic activity reduction was caused by changes in protein structure we performed CD spectroscopy.

### Structural analysis by CD spectroscopy

**Fig. 6:**
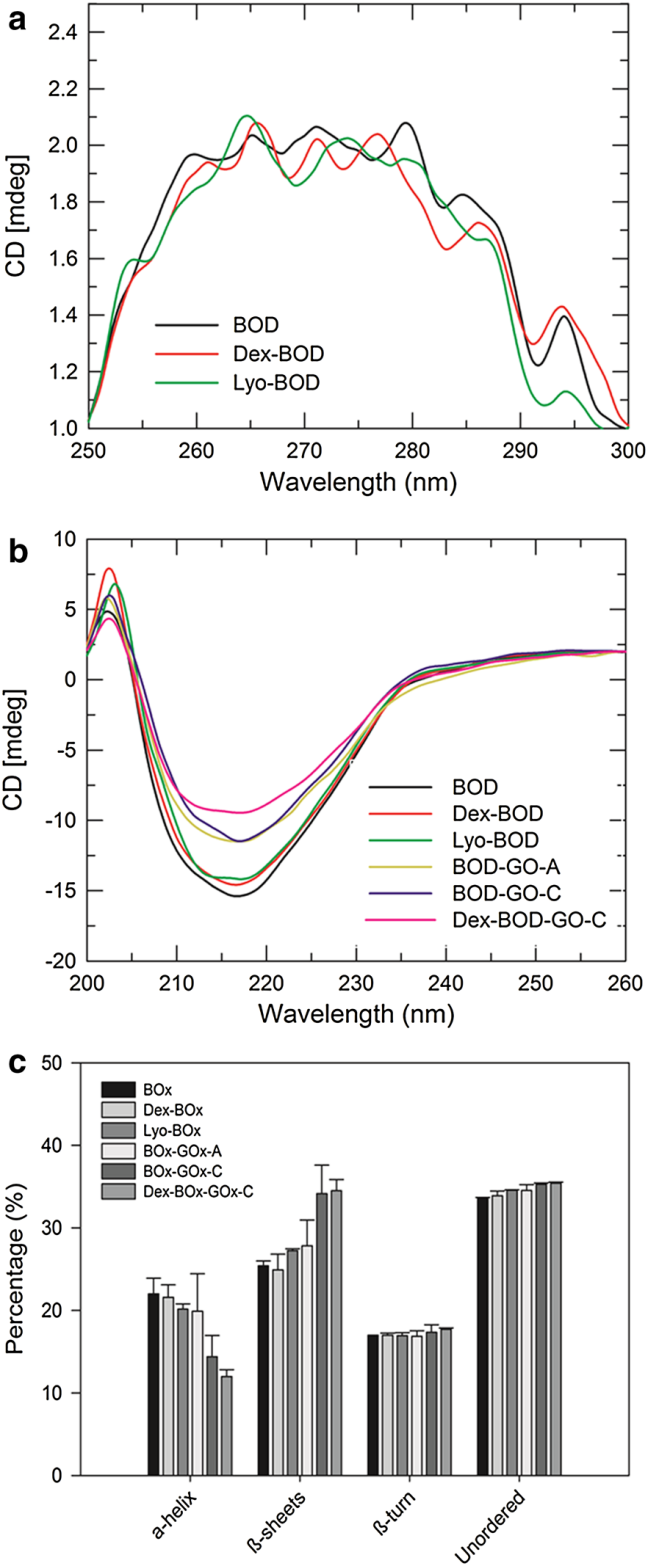
Near-UV CD spectra of BOD, Dex-BOD, Lyo-BOD in 0.1 M PBS pH 7.4 at 25 °C (**a**). Far-UV CD spectra of BOD, Dex-BOD, Lyo-BOD, BOD-GO-A, BOD-GO-C, and Dex-BOD-GO-C in 0.1 M PBS pH 7.4 at 25 °C (**b**). Estimates of the secondary structure calculated from the CD spectra using CDNN program (**c**). The data represented here was obtained by averaging three independent measurements (n = 3). The *error bar* represents the standard deviation

The CD spectra of proteins are dependent on their conformation; therefore, it is an excellent method to monitor conformational changes due to chemical glycosylation or binding interactions with nanomaterials. To assess the effect of the lyophilization and glycosylation process on the tertiary protein structure, we collected the CD spectra in the near-UV region (Fig. 6a). The spectrum of native BOD had six maxima between 250 and 300 nm that result from the environment of aromatic amino acids Phe, Tyr, and Trp [36]. The CD spectra of BOD, Dex-BOD, and Lyo-BOD were quite similar. In the far UV-region, BOD showed a negative CD band at 218 nm and a positive band at 205 nm, indicating the predominance of the β-structure (Fig. 6b) [40]. The intensities of the positive band in BOD-GO-A, BOD-GO-C, and Dex-BOD-GO were found to increase whereas the negative band decreased, in comparison with that of the native BOD. These results suggest a minimum change in the secondary structure of the BOD as a consequence of the interaction with GO. The CD spectra in the far UV region were analyzed using the CDNN program to obtain information about the secondary structural composition (Fig. 6c). These results indicate that the native conformation of BOD contains ~20 % α-helix, ~28 % of β-sheets, ~17 % β -turns and ~35 % of unordered structure, in agreement with previously reported data [36]. After the immobilization with GO, the amount of α-helix structure decreases and the amount of β-sheets increases, while the percentage of β–turns and unordered structure almost remains constant. These changes in the secondary structure as a result of the interaction with the GO may contribute to the loss in the catalytic activity of the protein. In contrast, the secondary structure of the chemically glycosylated BOD remained very similar to the native protein, suggesting that glycosylation helps stabilize protein structure.

### Protein structural dynamics: H/D exchange

Amide I (1600–1700 cm^−1^) region in protein spectra largely arise from C=O stretching vibration with a minor contribution from the C–N stretching vibrations and amide II (1500–1600 cm^−1^) largely results from N–H bending and C–N stretching vibrations [41]. Upon exposure to D_2_O, the amide II band shifts to lower wavenumbers (1400–1500 cm^−1^) as a result of the exchange with deuterium. Observing the decay of amide II intensity scaled by amide I intensity allows studying the protein dynamics as a consequence of glycosylation and immobilization on GO. The initial exchange rate is very fast since there are solvent exposed amide bonds. However, the amide bonds that are inaccessible to the solvent will exchange at a much slower rate. Therefore, the exchange rates of amide hydrogens that are less accessible or buried inside the native protein, provide a method for monitoring protein conformational changes and dynamical processes [42].

**Fig. 7:**
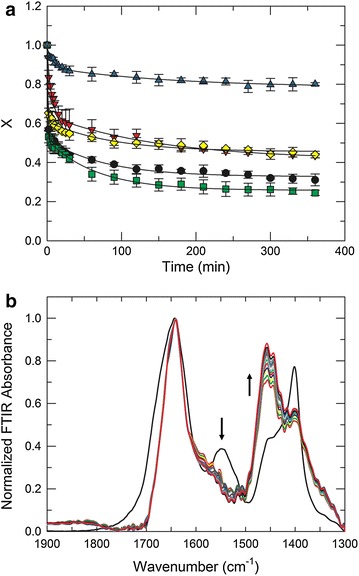
A typical FTIR-H/D exchange experiment for BOD, *arrows* highlight both the decreasing amide II band (N–H; 1550 cm^−1^) and the increasing amide II’ band (N–D; 1450 cm^−1^). **a** H/D exchange decay plots at 25 °C (pD 8.2) for BOD (*filled circle*), Dex-BOD (*filled inverted triangle*), BOD-GO-A (*filled square*), BOD-GO-C (*filled diamond*) and Dex-BOD-GO-C (*filled triangle*) (**b**)

Figure 7a shows the spectroscopic results from a typical FT-IR H/D exchange experiment for BOD, where the unexchanged amide groups (N–H) decreased while the exchanged ones (N–D) increased over time [43]. To examine the relationship between protein structural dynamics and amide hydrogen exchange, we plotted the fraction X of unexchanged amide groups over time (Fig. 7b).

Quantitative analysis of the decay plots was done with a bi-exponential model: [7, 43]

1 $$X = A_{1} exp^{{( - k_{HX,1} )t}} + A_{2} exp^{{( - k_{HX,2} )t}} + A_{3}$$

where A_1_, A_2_, and A_3_ are the fraction of the fast, slow and stable amide groups and k_HX,1_ and k _HX,2_ are the apparent exchange rate constants (Table 3). The decay data reveal that upon chemical glycosylation both, Dex-BOD and Dex-BOD-GO-C, showed a decreased in population (A_1_, A_2_) and rate constant (k_HX,1_, k_HX,2_) that correspond to the fast and slow exchanging amide groups, with an increase in the population of stable amide groups (A_3_). The observed stabilization of the enzyme conformation by protein glycosylation is consistent with other studies previously reported [7, 8]. We also found that the adsorbed bioconjugates (BOD-GO-A) exhibited a similar behavior when compared to the native protein, however a decrease in both rate constants (fast and slow) was obtained from the covalent bioconjugates (BOD-GO-C). These results suggest that the covalent immobilization of BOD in GO produced a reduction in protein dynamics by virtue of the covalent bond, while the adsorbed protein had a more dynamical structure. The confinement produced by GO can also modulate the biophysical and structural features of proteins [44]. This confinement can also be found inside the cell, where chaperonins produce a “cage effect” to facilitating protein folding, thereby helping them to reach their functional conformation [45]. Several computational studies have also shown that this “cage effect” produced protein stability and this stabilization increased with increasing confinement [44–46].

**Table 3:** Kinetic parameter derived from the H/D kinetic analysis

	A_1_	*K*_HX,1_ (min^−1^)	A_2_	*K*_HX,2_ (min^−1^)	A_3_	ΔG^mic^ (Kcal mol^−1^)	(ΔG^mic^)^−1^ (Kcal^−1^ mol)
BOD	0.479	1.062	0.192	0.0132	0.328	6.8	0.1472
Dex-BOD	0.336	0.1857	0.228	0.0067	0.415	940.1	0.0011
BOD-GO-A	0.483	1.049	0.259	0.0152	0.258	12.4	0.0805
BOD-GO-C	0.389	0.9721	0.155	0.0117	0.455	54.9	0.0182
Dex-BOD-GO-C	0.101	0.1257	0.104	0.006	0.783	1145.7	0.0009

The microscopic global free energy (ΔG^mic^) was calculated to gather information about which state was more favorable and its inverse (ΔG^mic^)^−1^ to know how protein mobility was affected. A substantial increase in ΔG^mic^ and a decrease in (ΔG^mic^)^−1^ were obtained from Dex-BOD and Dex-BOD-GO bioconjugates. This result indicates that the folded state of the protein was more favorable and protein mobility decreased due to an increase in protein stability conferred by the glycan attached to the protein. Studies have been shown that confinement enhances the stability due to the decrease in the entropy of the unfolded state and increases the stability of the native structure [44, 46]. The BOD-GO-A shows a slight increase in ΔG^mic^ for BOD-GO-C and a decrease in (ΔG^mic^)^−1^. In conclusion, the reduction in protein dynamics mostly depends on the chemical glycosylation of the protein, but also has a contribution that arises from the confinement produced by the immobilization on GO. These results shed some light on the reasons for the loss of specific activity that was obtained for Dex-BOD and Dex-BOD-GO-C, demonstrating that the activity loss is related to a decrease in protein dynamics and not due to protein unfolding.

### Thermal inactivation

**Fig. 8:**
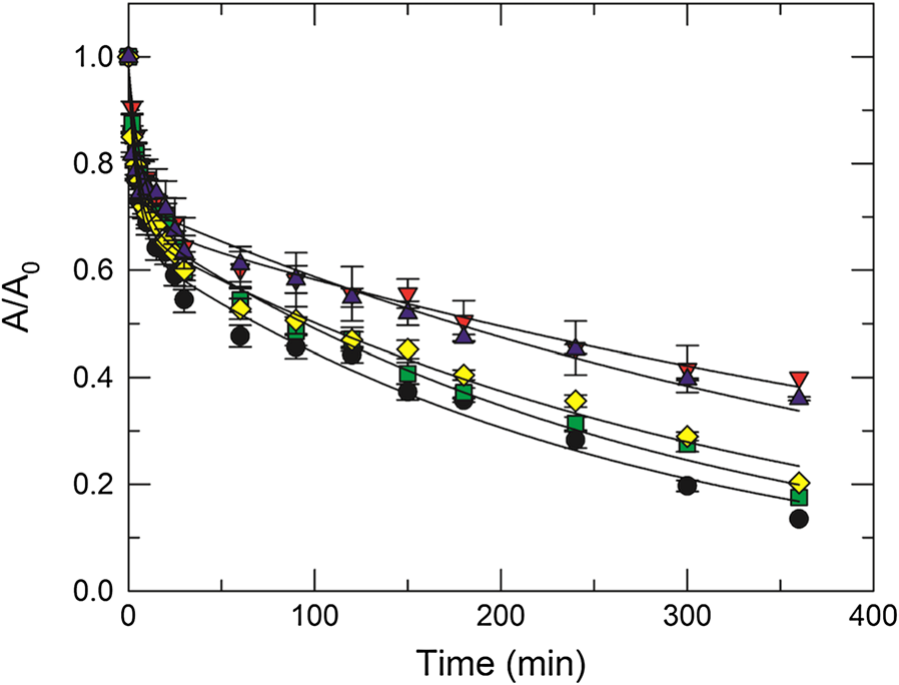
Kinetics of thermoinactivation at 45 °C for BOD (*filled circle*), Dex-BOD (*filled inverted triangle*), BOD-GO-A (*filled square*), BOD-GO-C (*filled diamond*) and Dex-BOD-GO-C (*filled triangle*) in 0.1 M PBS pH 7.4

In order to understand the influence of glycosylation and GO on protein thermostability we measured the enzyme kinetics as a function of time at 45 °C (Fig. 8). The thermal inactivation process can be fitted using an exponential decay model that consists of two kinetic rate constants; k_1_ (fast) and k_2_ (slow). These inactivation processes are described by the following equation: [7]

2 $$A/A_{0} = \alpha_{1} exp^{{( - k_{1} t)}} + \alpha_{2} exp^{{( - k_{2} t)}}$$

where A/A_0_ is the relative enzymatic activity and α_i_ and k_i_ represent the populations and the rate constants for the two inactivation processes. We found that glycosylation reduced both kinetic constants (k_1_ and k_2_) and increased the half-life (t_1/2_) for the fast and slow inactivation processes in Dex-BOD and Dex-BOD-GO-C (Table 4**)**. It has been shown that glycosylation reduces the protein solvent accessible surface area by sterically shielding the surface of the protein [47]. This shielding produces an increase the internal non-covalent binding forces and simultaneously leads to a decrease in protein structural dynamics, thereby increasing the protein thermodynamic stability [47]. BOD-GO-C showed a similar behavior, but not as notable as glycosylated samples. These results confirmed that glycosylation produced a significant increase in the functional half-life of the glycoconjugate and glyco-bioconjugate even at elevated temperatures. We also found that BOD-GO-A behaves similar to the native protein which could be attributed to the fact that at higher temperatures a desorption and adsorption equilibrium may be occurring in solution. This could cause the adsorbed protein to lose its catalytic activity faster than the covalently attached protein. The inactivation process of the covalently immobilized protein (BOD-GO-C) may be slower because GO provides a confinement restricting enzyme’s mobility; as a result the enzyme retains the tertiary structure for longer, when compared to BOD-GO-A. These results are consistent with the findings of H/D exchange experiments.

**Table 4:** Kinetic parameters derived from the thermal inactivation experiment

	α_1_	*K*_1_ (min^−1^)	t_1/2_ (min)	α_2_	*K*_2_ (min^−1^)	t_1/2_ (min)
BOD	0.31 ± 0.02	0.19 ± 0.03	3.6	0.67 ± 0.01	0.0029 ± 0.0002	239.0
Dex-BOD	0.69 ± 0.01	0.13 ± 0.02	5.3	0.30 ± 0.02	0.0016 ± 0.0001	433.2
BOD-GO-A	0.29 ± 0.02	0.18 ± 0.03	3.9	0.69 ± 0.1	0.0035 ± 0.0002	198.0
BOD-GO-C	0.33 ± 0.02	0.20 ± 0.03	3.5	0.65 ± 0.02	0.0038 ± 0.0002	182.4
Dex-BOD-GO-C	0.73 ± 0.03	0.15 ± 0.09	4.6	0.26 ± 0.01	0.0016 ± 0.0002	330.7

## Conclusions

The primary goal of this research was to determine if chemical glycosylation increases the stability of BOD in solution and immobilized on GO nanosheets. We also wanted to determine the mechanism by which glycosylation increases stability and how GO influences the bioconjugates stability. The results obtained demonstrate that chemical glycosylation of BOD induces a decrease in catalytic activity that was unrelated to conformational changes or a loss in binding substrate affinity. By measuring H/D exchange we found that after glycosylation the kinetics of H/D exchange are indeed reduced, confirming that the loss in activity was related to a reduction in protein structural dynamics. A decrease in protein dynamic and an increase in thermostability were observed for the covalent bioconjugate, when compared to BOD-GO-A. This thermostability improvement is caused by the confinement induced by the close proximity to GO nanosheets and the lack of freedom due to the covalent immobilization of the protein. When we used the chemically glycosylated protein to form the covalent bioconjugates (Dex-BOD-GO-C), we found a decrease in catalytic activity that was produced by the glycosylation and not by the immobilization process, since the specific activity results were similar. A drastic decrease in protein dynamics was found, creating an even higher thermostable bioconjugate. This is a consequence of the confinement produced by the glycans and GO nanosheets. In conclusion, we found that glycosylation helps producing a highly stable protein-GO bioconjugate for bio-nanotechnological applications, by restricting protein mobility and thus increasing stability.

## Methods

### Chemicals

Bilirubin oxidase (2.60 U mg^−1^) from *Myrothecium sp.* was donated from Amano Enzyme Inc. (Elgin, IL). Graphite platelet nanofibers 98 % (50–250 nm), 2,2′-azino-bis (3-ethylbenzothiazoline-6-sulphonic acid) (ABTS) ≥98 %, potassium permanganate (KMnO_4_) ≥99 %, and 30 % hydrogen peroxide (H_2_O_2_) were purchased from Sigma-Aldrich (St. Louis, MO, USA). Sulfuric acid (H_2_SO_4_) (OPTIMA™) and sodium phosphate monobasic 99 % (Acros) were from Thermo Scientific (Fair Lawn, NJ, USA). Dextran hexanoic acid (Dex-COOH, M_w_ 1 kDa) was purchased from Carbomer (San Diego, CA, USA). 1-Ethyl-3-[3-dimethylaminopropyl] carbodiimide hydrochloride and *N*-hydroxysulfosuccinimide (99 %) were from Proteochem (Denver, CO, USA).

### Glycosylation of BOD with dextran

First, the carboxylic acid group of dextran hexanoic acid was activated with 20 mM of EDC/25 mM of sulfo-NHS in 0.1 M MES, 0.5 M NaCl at pH 6.0 for 30 min at room temperature. Afterwards, EDC/sulfo-NHS was removed by dialysis (M_w_ cut-off of 100–500 Da) for 3 h against 0.1 M MES, 0.5 M NaCl at pH 6.0 and then for 2 h against nanopure water to remove salts, because the intermediated is stable at pH 7 for 4–5 h. Then sulfo-NHS-dextran was frozen in liquid N_2_ and lyophilized for 48 h. Chemical protein glycosylation of BOD (20 mg mL^−1^) was achieved by the addition of sulfo-NHS-dextran at a molar ratio of 1:6 (mol protein: mol dextran) in 100 mM PBS, 150 mM NaCl at pH 7.2 for 3 h. Reaction mixtures were gently stirred at 4 °C for 3 h followed by dialysis purification (M_w_ cut-off 20 kDa) for 24 h at 4 °C, flash frozen with liquid N_2_, and lyophilization. The degree of protein modification was determined by colorimetric titration of unreacted amino groups with 2,4,6-trinitrobenzene sulfonic acid [37].

### Preparation of bilirubin oxidase-graphene oxide bioconjugates

Bilirubin oxidase-graphene oxide bioconjugates (BOD-GO) were immobilized following two different methodologies: by adsorption and covalently linked. The adsorbed bioconjugates were prepared by mixing BOD (0.5 mg mL^−1^) with GO (0.1 mg mL^−1^) at 4 °C for 30 min. The mixture was then centrifuged at 5000*g* for 15 min using a centrifugal filtering unit (M_w_ cut-off 100 kDa). The supernatant was collected to determine the enzyme loading. The adsorbed bioconjugates (denoted as BOD-GO-A) were rinsed three times with water to remove nonspecifically adsorbed enzyme. Then, they were resuspended in water, frozen in liquid N_2_, and lyophilized for 48 h. To prepare the covalent biocongugates, denoted as BOD-GO-C for the native and Dex-BOD-GO-C for the glycosylated protein, we first activated the carboxylic groups at the surface of GO. The activation was accomplished by mixing GO (0.1 mg mL^−1^) with 20 mM of EDC/25 mM of sulfo-NHS in 0.1 M MES, 0.5 M NaCl at pH 6.0 for 30 min at room temperature. Then, EDC/sulfo-NHS was removed by repeated washing with nanopure water using the centrifugal filtering unit (M_w_ cut-off 100 kDa) until the absorbance (280 nm) in the supernatant was zero. Sulfo-NHS-GO (0.1 mg mL^−1^) was dissolved in buffer and mixed with the BOD (0.5 mg mL^−1^) or Dex-BOD (0.5 mg mL^−1^) for 3 h at 4 °C to obtain the covalent bioconjugate and glyco-bioconjugate. Then, we followed the same purification procedure as described for the adsorbed bioconjugates.

### Activity assays

The specific activity of BOD and the bioconjugates was determined photometrically by monitoring the oxidation reaction of 2,2′-azino-bis(3-ethylbenzthiazoline-6-sulphonic acid) (ABTS) at 340 nm (ε340 = 3.45 × 10^−4^ M^−1^ cm^−1^) on a Shimadzu 2450 UV/Vis spectrophotometer. The reactions were carried out in 0.1 M PBS pH 7.4 and started by the addition of 150 μl of enzyme ([E_0_] = 30 nM) to 100 μl of ABTS ([S_0_] = 10 mM) in a final volume of 1 ml at 25 °C. The kinetic parameters were determined from initial velocities using five substrate concentrations ranging from 1 to 20 mM. Lineweaver–Burk plot analysis was used to determine the Michaelis–Menten parameters *K*_*m*_ and *k*_cat_. For the thermal inactivation experiments, protein samples were incubated at 45 °C for 2, 4, 6, 8, 10, 15, 20, 25, 30 min, and then every 30 min for a period of 6 h. Then, the residual activity was assayed at 25 °C as described above.

### Characterization of GO and BOD-GO bioconjugates

The X-ray photoelectron spectra (XPS) recorded on a Physical Electronics Quantum 2000 Scanning ESCA Microscope spectrometer with an Al K X-ray source at 15 kV and 25 W. The pass energy used was 117.40 eV for the survey analysis and 58.70 eV for the high-resolution energy studies. Binding energies were corrected with respect to the aliphatic hydrocarbon C1s signal at 284.5 eV [48]. Raman spectra were recorded on a Thermo Scientific DXR Raman Microscope with a laser source of 532 nm, 20X objective, and 9 mW laser power; an average of 120 scans were recorded for each sample. FT-IR spectra were measured on a Nicolet NEXUS 470 infrared spectrophotometer from 400 to 4000 cm^−1^. Samples were prepared as KBr pellet. Spectra were recorded in transmission mode at 2 cm^−1^ and 240 scans were averaged to obtain each spectrum. The XRD spectra were measured on a Rigaku Smart Lab with and Cu Ka X-ray source (λ = 1.54 Å) and Kb filter. Zeta-potential values were recorded on a Zetasizer Nanoseries (Malvern).

### Circular dichroism (CD) spectroscopy

CD spectra were acquired with a JASCO J-1500 CD spectrophotometer at 25 °C. BOD, Lyo-BOD, Dex-BOD, and BOD-GO bioconjugates were prepared in 0.1 M PBS pH 7.4. CD spectra were recorded from 250 to 300 nm using 10 mm path length quartz cells for the tertiary structure and from 200 to 260 nm using 1 mm path length quartz cells for the secondary structure at a scan rate of 50 nm min^−1^ with a protein concentration of 0.6 and 0.2 mg mL^−1^, respectively. Each spectrum was obtained by averaging three scans at 1 nm resolution and solvent reference spectra were digitally subtracted from protein CD spectra. The changes in the secondary structures of the proteins and bioconjugates, in terms of α-helix, β-sheet, β-turn, and unordered structure, were calculated from the CD data using the CDNN program (version 2.0). Results presented for the changes in the secondary structure are an average of three solutions prepared independently.

### Kinetics of hydrogen/deuterium (H/D) exchange

Amide H/D exchange FTIR spectra were measured on a Nicolet NEXUS 470 infrared spectrophotometer using CaF_2_ windows and 25 μm Teflon spacers. Exchange time was started at the moment of D_2_O addition. Spectra (2000–1300 cm^−1^) were collected at 2, 4, 6, 8, 10, 15, 20, 25, and 30 min, then every 30 min for a period of 6 h. For the first 30 min, 24 scans were averaged, and after 30 min, 60 scans, all at 2 cm^−1^ resolution. Spectra of buffer blanks in D_2_O were subtracted from all sample spectra. Samples were prepared at a concentration of 4 mg mL^−1^, and 1 mL was flash frozen with liquid N_2_ followed by lyophilization. Samples were dissolved in 200 μl of D_2_O yielding a final protein concentration in the cell of 40 mg mL^−1^.

H/D exchange spectra were processed for quantitative analysis in the form of hydrogen exchange decay plots (X vs. time). The fraction X of unexchanged backbone hydrogen atoms at time t was determined by the following equation: [7, 49]

3 $$X = [w\left( t \right) - w\left( \infty \right)]/[w\left( 0 \right) - w\left( \infty \right)]$$

where w(t) is the ratio of the baseline corrected absorbance of amide II (1544 cm^−1^) to amide I (1640 cm^−1^) at a time *t*, w(0) is the amide II/amide I ratio of the undeuterated proteins and w(∞) is the amide II/amide I ratio for the fully deuterated proteins. w(0) was calculated from IR spectra for the undeuterated proteins measured as KBr pellets and w(∞) from samples incubated for 15 days in D_2_O at 37 °C to ensure complete exchange. The pH values of the H/D exchange experiments were taken from the direct readings observed on the pH meter and the pD values were calculated using the following equation: pD = pH + 0.4 [50]. Experimental pD values were measured from samples after the addition of D_2_O and were found to be 8.2.

The global Gibbs free-energy of microscopic unfolding (ΔG^*mic*^) per mole of peptide was calculated through thermodynamic analysis of the H/D exchange kinetics using the following equation: [7, 50]

4 $$\Delta G^{mic} = - RT(k_{obs} /k_{0} )$$

where k_obs_ = k_HX,1_ + k_HX,2_ known as the measured rate constants, and k_0_ is the chemical exchange rate constant. The chemical exchange rate constant (k_0_) was calculated as a function of temperature and pH according to the following equation: [50]

5 $$k_{0} = \left( {10^{ - pH\,\, read} + 10^{ - pH\,\, read - 5} } \right)10^{{0.05\left( {T - 25} \right)}} s^{ - 1}$$

## Additional file

10.1186/s12951-015-0134-0 In the Supplemental Material Section the synthesis and characterization of GO are shown.

## Abbreviations

GO: graphene oxide
BOD: bilirubin oxidase
FT-IR: fourier transform infrared spectroscopy
XPS: X-ray photoelectron spectroscopy
CD: circular dichroism
*K*_*m*_: Michaelis–Menten constant
*k*_*cat*_: turnover number
PEG: polyethylene glycol
NHS-tripod: *N*-hydroxysuccinimidyl ester tripod
Con A: concanavalin A
GOD: glucose oxidase
HRP: horseradish peroxidase
OxOx: oxalate oxidase
Cyt-c: cytochrome c
BSA: bovine serum albumin
Dex-BOD: glycosylated bilirubin oxidase
Lyo-BOD: lyophilized bilirubin oxidase
BOD-GO-A: adsorbed bilirubin oxidase on graphene oxide
BOD-GO-C: covalent attached bilirubin oxidase on graphene oxide
Dex-BOD-GO-C: glycosylated bilirubin oxidase covalent attached on graphene oxide
ΔG^mic^: microscopic global free energy
(ΔG^mic^)^−1^: protein mobility
