# Supplementary material for: Graphene oxide as a protein matrix: influence on protein biophysical properties
Source: J Nanobiotechnology. 2015 Oct 19;13:70. doi: 10.1186/s12951-015-0134-0 (PMC4617716; doi:10.1186/s12951-015-0134-0)
Supplement: Supplementary file 1 — 10.1186/s12951-015-0134-0 In the Supplemental Material Section the synthesis and characterization of GO are shown. [file 12951_2015_134_MOESM1_ESM.docx]

Supporting Information

**Graphene Oxide as a Protein Matrix: Influence on Protein Biophysical Properties**

Author(s), and Corresponding Author(s)*:

Griselle Hernández-Cancel, Dámaris Suazo-Dávila, Axel J. Ojeda-Cruzado, Desiree García-Torres, Carlos R. Cabrera, and Kai Griebenow*

Address:

Department of Chemistry, University of Puerto Rico, Río Piedras Campus, San Juan P.R. 00931, USA.

**Synthesis of Graphene Oxide Nanosheets**

GO nanosheets were prepared from graphite platelet nanofibers (GPNF) by a procedure adopted from L. Cunci *et al.*^1^ Briefly, graphite platelet nanofibers (100 mg) were dispersed by sonication in 40 mL of a mixture of concentrated H_2_SO_4_/H_2_PO_4_ (9:1) and, 600 mg of KMnO_4_ was slowly added to the mixture under constant stirring and left for 12 h at 50 °C. Reaction time was less than the one reported by L. Cunci to maintain the electrical properties of GO, because as the oxidation increases the material becomes less conductive. Afterwards, the reaction was cooled to room temperature and 400 mL of ice-cold nanopure water was poured slowly into the solution under vigorous stirring. The suspension was treated further by dropwise addition of 6 mL of 30% H_2_O_2_ until the solution turned bright yellow. The solution was heated to boiling, which turned the solution brown again. Afterwards it was filtered with a 0.8 μm Nylon filter. After filtration, the solid was dispersed in a mixture of water and DMF and centrifuged at 3400 rpm for 8 min. The supernatant was extracted, dispersed in abundant distilled water until the pH reached 7, and filtered with a 0.1 μm pore size polycarbonate filter. The filtered material was lyophilized and ground into a fine powder.

**Characterization of Graphene Oxide Nanosheets**

The formation of GO nanosheets was monitored using FT-IR spectroscopy (Figure S1A). After the oxidation procedure, peaks appeared at frequencies corresponding to oxygen functionalities, such as carbonyl stretching (C=O) at 1720 cm^-1^, stretching vibration of O-H groups at 3395 cm^-1^ and bending vibration of C-O-H at 1380 cm^-1^, C-O from epoxy groups at 1226 cm^-1^, and C-O from alkoxy groups at 1080 cm^-1^.^2^ The peak at 1640 cm^-1^ corresponds to skeletal vibrations of unoxidized graphitic domains (C=C). ^3^

The X-ray diffraction (XRD) spectra (Figure S1B) support that GPNF was oxidized to form GO. The interlayer spacing of the material is proportional to the degree of oxidation. Bragg’s law was used to calculate the distance (d) between the atoms. This law states that nλ = 2d sinθ, where n is an integer, λ the wavelength, d is the spacing between the layers of atoms, and θ is the angle between the layers.^4^ GPNF shows a characteristic sharp (002) peak at 29.59° that corresponds to a value of interplanar spacing of 0.34 nm. The oxidation process produced an increase in the interlayer distance that results in a shift of the (002) peak to a lower angle with a broad peak at 9.24°, which corresponds to an interplanar distance of 0.96 nm in GO.

The GO formation was also confirmed by Raman spectroscopy (Figure S1C). The variation of the relative intensities of G and D bands in Raman spectra usually reveal changes in the electronic conjugation state of the GPNF. The D band is due to the breathing mode of sp^2^ atoms in the rings and its intensity is associated to the amount of defects.^5^ The G-band represents the planar configuration sp^2^ bonded carbon; the position of the band and its shape provides information on the layer thickness. As the layer thickness decreases, the G-band position shifts to higher frequencies.^6^ The Raman spectra of GPNF contain a G band at 1568.26 cm^-1^ and a D band at 1338.83 cm^-1^, and the second-order bands at 2672 cm^-1^ (2D) and 2909.71 cm^-1^ (D+G), as previously reported.^7^ After the oxidation, the G band broadened significantly and displayed a shift to higher frequencies (1590.57 cm^-1^) and the D band (1346.01 cm^-1^) grew in intensity. Those changes suggest that the amount of layers in GO was reduced and oxygen functionalities were introduced leading to an increase in defects in agreement with previously reported data.^6,8^ A significant change in the Raman spectra of GO in terms of shape and intensity of the 2D band was observed compared to GPNF. When graphene has less than 5 layers, double or multiple 2D bands can be distinguished.^9^ Thus, the shift of the G band, the relatively intense D band, and the change in the shape and intensity of the 2D band indicate small stacks of quite disordered GO nanosheets.

The oxidation progress was also monitored by UV-Vis spectroscopy (Figure S1D). Both materials were prepared at a concentration of 0.05 mg mL^-1^ in water and dispersed by sonication for 15 min. The absorption intensity is basically constant over the entire spectrum as result of the reduced form of GPNF, with a minor peak at 252 nm. After the oxidation, GO exhibits an absorption band at 226 nm that corresponds to the π-π* transition of aromatic C=C bonds, and a shoulder at 303 nm due to n–π* transition from the C=O bond.^10^ The absorption intensity of the entire spectrum of the GO, especially in the region above 300 nm, is similar to GPNF, suggesting that the GO is partially oxidized. The color of the GPNF changes from black to yellowish brown as result of the oxidation indicating that the formation of GO was successful (Figure S1D, inset).

Zeta-potential measurements can provide additional evidence of the oxidation process. Table S1 shows the average zeta-potential values obtained for GPNF and GO, both prepared at a concentration of 0.1 mg mL^-1^ in nanopure water. The zeta-potential increased from -6 mV to -47 mV owing it to the oxidation process. GPNF has a slightly negative charge indicating the presence of a limited amount of oxygen moieties. This result supports the fact that a D band that corresponds to defects in the material can be observed in the Raman spectra as well as O-H band in the FT-IR spectra of GPNF.


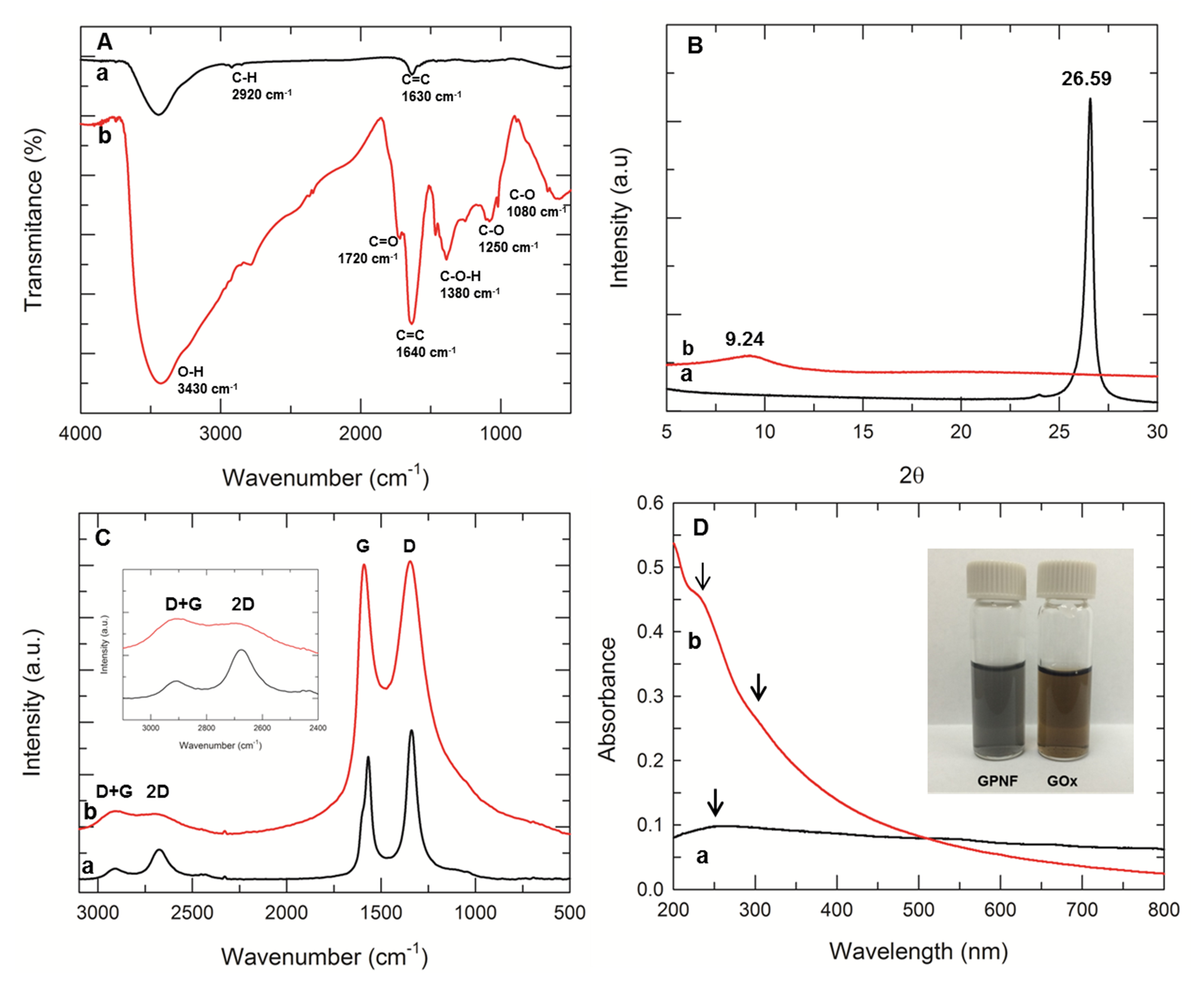


**Figure S1. FT-IR spectra (A), X-ray diffraction spectra (B), Raman spectra (C), UV-Vis spectra (D), and photograph (D, inset) of GPNF (a) and GO (b).**

**Table S1. Zeta-potential values of graphene oxide after the oxidation process.**

| Sample | Solvent | Z Potential |
| --- | --- | --- |
| GPNF | Water | -6 ± 1 |
| GO |  | -47 ± 1 |

References

1. Cunci, L.; Velez, C. A.; Perez, I.; Suleiman, A.; Larios, E.; Jose-Yacaman, M.; Watkins, J. J.; Cabrera, C. R., Platinum electrodeposition at unsupported electrochemically reduced nanographene oxide for enhanced ammonia oxidation. *ACS Appl Mater Interfaces* **2014,** *6*, 2137-45.

2. Zhang, J.; Yang, H.; Shen, G.; Cheng, P.; Zhang, J.; Guo, S., Reduction of graphene oxide via L-ascorbic acid. *Chem Commun (Camb)* **2010,** *46*, 1112-4.

3. Si, Y.; Samulski, E. T., Synthesis of water soluble graphene. *Nano Lett* **2008,** *8*, 1679-82.

4. Shao, Y.; Zhang, S.; Wang, C.; Nie, Z.; Liu, J.; Wang, Y.; Lin, Y., Highly durable graphene nanoplatelets supported Pt nanocatalysts for oxygen reduction. *Journal of Power Sources* **2010,** *195*, 4600-4605.

5. Ferrari, A. C., Raman spectroscopy of graphene and graphite: Disorder, electron–phonon coupling, doping and nonadiabatic effects. *Solid State Communications* **2007,** *143*, 47-57.

6. Srinivas, G.; Zhu, Y.; Piner, R.; Skipper, N.; Ellerby, M.; Ruoff, R., Synthesis of graphene-like nanosheets and their hydrogen adsorption capacity. *Carbon* **2010,** *48*, 630-635.

7. Chu, P. K.; Li, L., Characterization of amorphous and nanocrystalline carbon films. *Materials Chemistry and Physics* **2006,** *96*, 253-277.

8. Zavodszky, P.; Johansen, J. T.; Hvidt, A., Hydrogen-exchange study of the conformational stability of human carbonic-anhydrase B and its metallocomplexes. *Eur J Biochem* **1975,** *56*, 67-72.

9. Kim, H. J.; Lee, S. M.; Oh, Y. S.; Yang, Y. H.; Lim, Y. S.; Yoon, D. H.; Lee, C.; Kim, J. Y.; Ruoff, R. S., Unoxidized graphene/alumina nanocomposite: fracture- and wear-resistance effects of graphene on alumina matrix. *Sci Rep* **2014,** *4*, 5176.

10. Yang, S.; Yue, W.; Huang, D.; Chen, C.; Lin, H.; Yang, X., A facile green strategy for rapid reduction of graphene oxide by metallic zinc. *RSC Advances* **2012,** *2*, 8827-8832
